# Climate for Consumptives

**Published:** 1870-12

**Authors:** 


### HALL'S

### JOURNAL OF HEALTH.

[*All Articles are written by the Editor.*]

VOL. XVII.—DECEMBER, 1870.—NO. XII.

### CLIMATE FOR CONSUMPTIVES.

The sooner a consumptive wants to die, let him go to Minnesota; hence the most intelligent and honorable physicians who have have lived there longest, advise the wanderers who are actually consumptive, and come there for restoration, to return home immediately.

Everybody knows that to be cooped up in a hot room all winter, is enough to kill a well man, and certainly it cannot restore to health a man who is already half dead with consumption.

A consumptive man is one who has already lost the use of a part of his lungs—the more need, then, is there that the air which he does consume should be the purest possible. No one will pretend that a close stove-room has a pure atmosphere.

Every consumptive of moderate intelligence and observation, knows very well that he cannot stand the cold; he shudders at the very thought of ice and snow, and hugs stove or chimney-corner with inexpressible delight.

There is not a consumptive in existence who will voluntarily leave the house when the thermometer is at forty or under. The healthiest of us shiver when we go out of doors in winter, and the air is near the freezing point of thirty-two degrees; but in Minnesota, in winter, the mercury is often ten, fifteen, and even twenty degrees below zero; that is, fifty or sixty degrees colder than above stated. Being out of doors, in such an atmosphere, is simply impossible to a consumptive—his lungs will begin to ache in half an hour—and there are many times when the stoutest men cannot be exposed an hour at a time.

These are plain facts which every man, sick or well, knows almost by intuition, that a consumptive cannot bear very cold weather, and is compelled to remain indoors, in a close, warmed room, the air of which must be vitiated. Hence, to go to Minnesota after the first of November to remain until Spring, as a means of benefitting a person who is really in consumption, is idiotic.

The out-door air is pure, is cold, is bracing, but only to those who breathe it for a great part of the twenty-four hours. If a man is in the first stages of consumption; that is, has not much shortness of breath, no night-sweats, no swollen feet, and has a pulse which is never over eighty-five beats in a minute when at rest for half an hour, and not within an hour after eating a regular meal; such a man, by being a great deal in the saddle, and is for a large part of daylight in the open air, working, hunting, fishing, botanizing, studying the rocks, prospecting for rich mines, or richer lands, for investment or settlement; such a man will revel in the delicious, pure and bracing air of that lovely country; he will fatten up and strengthen every day; every day he can eat more and more, will cough less and less, will sleep sounder, walk more blithely, and whistle and sing louder.

But with any one of the symptoms above enumerated, in connection with a cough, more or less present every day, a Minnesota climate for a single winter is certain death.

The weather in Minnesota is balmy and pleasant for many days in winter—a day or two or three at a time; but even then there is a heavy dampness in the air, and there is a great deal of violent, fierce, cold wind, that seems almost to cut a healthy man in two.

March and April are, as a general rule, so insufferably disagreeable, that many arrange to leave the country.

At times the air is dry in winter; but, if the least thing is the matter with the throat, it makes it so raw in half an hour, that it seems to be all on fire. If one has throat disease, a visit to Minnesota for the winter will be fatal, unless he stays in the house; that he could do at home.

The climate for ordinary consumptives who are determined to leave home, with all its attachments and comfortable surroundings, to be exchanged for hireling attendance, for the desolations of loneliness, and the thousand-and-one discomforts which fret, and worry, and annoy the hapless invalid among strangers, and in a strange country, the climate best adapted for such should be uniform in its temperature, and moderate enough to allow an exposure to the out-door air for the greater part of the twenty-four hours. Aiken, South Carolina, has many advantages, but we do not advise any one to go there, because a tavern-keeper at that place never could be induced to respond to a bill of advertising in the *Journal.* If the climate makes people mean, they had better not go there.

Florida is too hot, and low, and damp—there is so little that is bracing in its atmosphere; and then its mosquitoes, and alligators, and bull frogs, its ticks, and its sand flies, are enough to deter the stoutest heart.

All things considered, the most enjoyable place away from home for the consumptive, is New Providence Island, in the West Indies. To its chief town Nassau, steamships run regularly from New York city. It is in latitude 25° west, longitude 77°. The average temperature of winter is seventy degrees, and of Spring seventy-seven. The coldest day in Nassau in twenty years was sixty-four degrees, and the warmest from November to May was eighty-two. Sometimes during March and April, there are not half a dozen rains, and these do not last more than two or three hours each, so that visitors can spend almost all the time during daylight in the open air; and for consumptives, out-door air for the greater part of twenty-four hours is essential to any radical improvement.

